# High ceruloplasmin levels are associated with obsessive compulsive disorder: a case control study

**DOI:** 10.1186/1744-9081-4-52

**Published:** 2008-11-18

**Authors:** Osman Virit, Salih Selek, Mahmut Bulut, Haluk Asuman Savas, Hakim Celik, Ozcan Erel, Hasan Herken

**Affiliations:** 1 Department of Psychiatry, Medical Faculty, Gaziantep University, Gaziantep, Turkey; 2 Department of Psychiatry, Kahramanmaras State Hospital, Kahramanmaras, Turkey; 3 Department of Clinical Biochemistry, Medical Faculty, Harran University, Sanliurfa, Turkey; 4 Department of Psychiatry, Medical Faculty, Pamukkale University, Denizli, Turkey

## Abstract

**Background:**

Alterations in ceruloplasmin are currently assumed as one of the mechanisms underlying the development of a number of neurodegenerative disorders. Several studies indicate that elevated serum ceruloplasmin levels may play a role in schizophrenia by exacerbating or perpetuating dopaminergic dysregulation. No study investigating the relationship between ceruloplasmin and obsessive-compulsive disorder (OCD) has been published to date. Nowadays OCD is increasingly speculated to be a different disorder than other anxiety disorders, and rather is considered to be more similar to psychotic disorders. The objective of this study to explore whether there is an association of ceruloplasmin with OCD as in schizophrenia.

**Method:**

26 pure OCD and 9 co-morbid OCD patients from Gaziantep University Sahinbey Research Hospital, Psychiatry Clinics, diagnosed according to the DSM IV and 40 healthy controls were included in the study. Blood samples were collected; ceruloplasmin levels were measured.

**Results:**

The mean ceruloplasmin level in pure OCD patients, co-morbid OCD patients, and control group persons were 544.46 ± 26.53, 424.43 ± 31.50 and 222.35 ± 8.88 U/L respectively. Results of all 3 groups differ significantly. Positive predictive value of ceruloplasmin for that cut-off point is 31/31 (100%) and negative predictive value is 40/44 (91%) in our group.

**Conclusion:**

Although the nature of relationship is not clear there was an association between ceruloplasmin levels and OCD in our study.

## Background

Ceruloplasmin is a protein of the α2-globulin fraction of human blood serum. It contains 95% of serum copper. Hepatocytes synthesize ceruloplasmin, which is subsequently found in plasma, but extrahepatic gene expression has been documented for this protein. Among the organs expressing ceruloplasmin gene are the brain, lung, spleen, and testis. In the central nervous system of humans and other mammals, ceruloplasmin is expressed in neurons and astroglial cells, e.g., of the cerebral microvascular network. Ceruloplasmin carries copper from liver to numerous tissues. The symptoms of Wilson's disease, that is characterized by low levels of ceruloplasmin with subsequent copper deposition in various tissues including brain, often mimic those of schizophrenia. Ceruloplasmin is also an iron oxidase; for this capacity, it has been labeled ferroxidase I. In addition, ceruloplasmin is an acute phase reactant, whose concentration increases in inflammation, infection, trauma, etc. For these properties it is known as an antioxidant. Ceruloplasmin has other functions, including the oxidation of serotonin, epinephrine, and norepinephrine. The relation of ceruloplasmin to neurodegenerative processes in human was documented. Alterations in ceruloplasmin levels are currently regarded as one of the mechanisms underlying the development of a number of neurodegenerative disorders. Vassiliev et al. (2005) discussed largely ceruloplasmin in neurodegenerative diseases [1].

There are numerous studies reporting an association between ceruloplasmin and schizophrenia, but the nature of the relationship remains unclear [2-9]. One possible explanation has been ceruloplasmin and copper relation since abnormalities in copper levels or metabolism may lead to dopaminergic dysregulation. In addition, ceruloplasmin has been associated with some clinical features of schizophrenia [2,4,5,8]. Some symptomatology of OCD resembles to schizophrenia. Also dopaminergic dysregulation in the certain areas of brain may play a role in OCD etiopathogenesis as in schizophrenia [10,11]. Nowadays, OCD is increasingly speculated to be a different disorder than other anxiety disorders. OCD is rather thought to be similar to psychotic disorders [12,13]. The objective of this study is to explore, whether there is a role of ceruloplasmin in OCD as in schizophrenia. Therefore, we investigated the possible association between serum ceruloplasmin levels and OCD. We have not identified any study on this issue in the literature prior to our study.

## Methods

### Patients

37 consecutive OCD patients, who were diagnosed based on DSM-IV (30 females, 7 males), and followed by Department of Psychiatry, Gaziantep University Hospital after they applied to Anxiety Disorders Unit, were enrolled after they provided informed consent. The diagnoses of OCD were established by one psychiatrist (SS). Nine of the 37 patients had psychiatric comorbidities. In addition to OCD, five of 9 suffered from other anxiety disorders, 2 from depression, 1 from schizoaffective disorder. The remaining one patient had more than two psychiatric disorders. Patients with comorbid diagnoses were included in the study if the other psychiatric conditions were in remission. Remission was defined as a score below or equal to two ("borderline mentally ill") on Clinical Global Impression (CGI)-Severity Scale [14]. All patients were on their OCD treatment provided naturalistically by the same psychiatrist. The data were collected within two months. Exclusion criteria were as follows: alcohol or substance dependence according to the DSM-IV, tardive dyskinesia related to neuroleptics, presence of severe organic condition, use of any antioxidant agent (i.e. vitamins E and C), presence of epilepsy or other severe neurological disorder which were previously found to be associated with oxidative status, presence of infectious disease, excessive obesity and insufficient sampling. Two patients were excluded due to insufficient sampling.

### Controls

The control group consisted of forty healthy volunteers who were recruited from university staff (30 females, 10 males). They were free of any medication for at least 6 weeks prior to blood sampling. None of the control subjects were alcohol consumer, heavy smoker, or had ever taken any psychotropic drugs. They had no history or family history of any psychiatric disorder. The controls were matched with the patients in regards to sex and age. All subjects signed written informed consent, which had been approved by the local ethics committee in accordance with the Declaration of Helsinki. Physical and neurological examinations were performed on each of the patients and controls. Only subjects with normal physical and neurological exams and without any exclusion criteria were admitted to the study.

### Instruments

#### Sociodemographic information form

All subjects were evaluated by a semi-structured questionnaire, which was developed in accordance with clinical criteria and available information sources. Gender, age, marital status, smoking habits, socioeconomic status, and duration of illness were recorded before venous blood sampling.

#### Yale-Brown Obsession Compulsion Scale

Yale-Brown Obsession Compulsion Scale in Turkish (Y-BOCS) is a scale that assesses the severity of OCD without focusing on the contents of obsession and compulsion [15]. It has ten items. Each item is assessed between 0 and 4 points by a clinician. Patients were rated by the same psychiatrist (SS) twice, on the venous sampling day for initial Y-BOCS evaluation and after two months for late Y-BOCS scores. During second evaluation the rater was blinded to the serum measures.

### Blood sampling

Venous blood samples from left forearm vein were collected into 5 ml vacutainer tubes between 7 and 8 a.m. after overnight fasting. The blood samples were centrifuged at 2000 rpm for 10 min to obtain sera. Samples were stored frozen at -40°C before analysis. The biochemical analyses were made after all the blood samples were collected.

### Ceruloplasmin measurement

Erel's ceruloplasmin measurement method is used. This method is automated, colorimetric, and based on the enzymatic oxidation of ferrous ion to ferric ion [16]. The results were expressed in milligrams per deciliter, and the precision of this assay is within 3%. For a details see Erel [16,17].

### Apparatus

A Cecil 3000 spectrophotometer with a temperature controlled cuvette holder (Cecil) and an Aeroset automated analyzer (Abbott) were used [16].

### Statistical Analysis

The obtained data were evaluated by SPSS for Windows 13.0. The comparisons were performed by ANOVA with Tukey HSD (for two-group and all-group comparisons). For correlation evaluations, the Spearman correlation (two-tailed) was used. The comparison of sociodemographic characteristics was performed by the independent samples T test. Two-tailed forms were used. The comparison of serum levels among groups was performed by Post Hoc Tukey's Test and Dunnett's Test. The statistical significance was accepted as p < 0.05. Using receiver operating characteristic (ROC) analysis, curve was plotted of sensitivity versus 1 minus specificity for all possible cut-off scores of serum ceruloplasmin level as a diagnostic test. The accuracy of the serum ceruloplasmin level as a diagnostic test in OCD is represented by, and is directly proportional to, the area under the curve (AUC).

## Results

**Figure 1:**
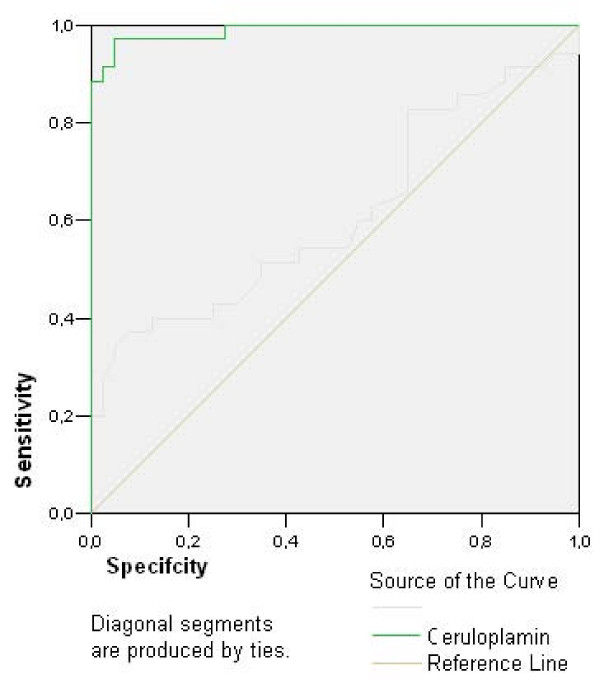
In plotted ROC curve, disease condition (OCD) can be predicted via ceruloplasmin levels over 342.85 U/L with 88.6% sensitivity and 100% specificity.

The demographic and clinical data of the subjects were summarized in Table 1. The mean ceruloplasmin levels in pure OCD patients, co-morbid OCD patients and control group persons were 544.46 ± 26.53, 424.43 ± 31.50 and 222.35 ± 8.88 U/L respectively. The mean ceruloplasmin levels were significantly higher in pure and co-morbid OCD groups than that of control group (p < 0.01). In pure OCD group mean ceruloplasmin level was significantly higher than co-morbid OCD group (p < 0.01). In plotted ROC curve (Figure 1) OCD could be predicted for ceruloplasmin over 342.85 U/L level with 88.6% sensitivity and 100% specificity (Table 2). Positive predictive value of ceruloplasmin for that cut-off point was 31/31 (100%) and negative predictive value was 40/44 (91%) in our study. There was no correlation between ceruloplasmin and illness duration or YBOCS scores (p > 0.05).

**Table 1:** Sociodemographic and clinical characteristics of patients

	All Patients	Pure OCD
Sex: female/male (n)	28/7	22/4
Age: Mean ± SD	33.4 ± 11.2	34.5 ± 11.0
Illness duration (years): Median	5 (1–30)	5 (1–30)
YBOCS-initial: Mean ± SD	9.8 ± 5.7	9.7 ± 5.8
YBOCS-later: Mean ± SD	9.2 ± 5.9	8.5 ± 6.0

**Table 2:** Characteristics and coordinates of ROC Curve

Variable	Area under Curve	P value	95% Confidence Interval	
			Lower Bound	Upper Bound
Ceruloplasmin	0.989	< 0.001	0.971	1.006
				
Coordinates of ROC curve				
				
Variable	Positive if greater or equal to	Sensitivity	Specificity	
				
Ceruloplasmin	327.8500	0.914	0.025	
	332.3500	0.886	0.025	
	342.8500	0.857	0.000	
	362.2500	0.829	0.000	
	376.9500	0.792	0.000	

## Discussion

The association between ceruloplasmin and OCD has not been investigated so far. To the best of our knowledge this is the first report about elevated ceruloplasmin levels in OCD patients. Ceruloplasmin abnormalities have been implicated in schizophrenia, even the nature of relationship remains unclear. Since abnormalities in copper metabolism may lead to dopaminergic dysregulation, the ceruloplasmin and copper relationship might be important in schizophrenia [9]. Ceruloplasmin largely determines copper concentration, and plays a critical role in the interpretation of copper results [18,19]. One suggested strategy to study copper is to measure ceruloplasmin levels, because approximately 95% of the copper in blood is bound to ceruloplasmin, therefore increases in ceruloplasmin would be expected to correlate directly with increases in serum copper. Both increased serum copper and ceruloplasmin levels have been found in the same schizophrenic patients [9]. The importance of dopamine has been largely studied in schizophrenia and copper plays role in the synthesis and metabolism pathways of dopamine. For example, the copper-dependent enzyme tyrosinase shunts tyrosine away from DOPA production, copper inhibits dopa-decarboxylase thereby inhibiting dopamine production, the copper-dependent enzyme dopamine beta-hydroxylase catalyzes the breakdown of dopamine into norepinephrine, and the copper-dependent enzyme monoamine oxidase (MAO) catalyzes the breakdown of dopamine into other metabolites. Elevated copper and corresponding changes in dopamine may result in increased number or sensitivity of post-synaptic dopamine receptors in certain areas of the brain. Thus, elevated ceruloplasmin results in increased copper, which may lead to psychotic symptoms through the mentioned mechanism. Similarly, psychotic symptoms occur in Wilson's disease, in which there is copper deposition in liver, cornea, and brain due to lack of ceruloplasmin. This leads to over exposure of brain to copper [9,20].

As in schizophrenia, association between ceruloplasmin and OCD might be examined through ceruloplasmin and copper relationship, since there is now growing evidence that the dopamine system may be involved also in the pathophysiology of OCD [21-23]. An association between OCD and increased midbrain dopamine neurotransmission has been reported. In addition, atypical antipsychotics, such as risperidone and quetiapine, may augment the response to selective serotonin reuptake inhibitors (SSRIs) in patients with refractory OCD. This would seem to point to an increase in dopaminergic system activity in OCD [24,25]. As in schizophrenia increased ceruloplasmin levels may increase copper levels, which may lead to dopaminergic dysregulation in OCD.

However, association between ceruloplasmin and OCD may be explained via copper and serotonin relationship, too. Copper induces oxidation of serotonin and the products of 5-HT oxidation are or have the potential to be neurotoxic, especially on serotonergic receptors. Copper structurally alters serotonin by effectively converting functional 5-HT into dimeric species. It has been suggested that this process might play a role in copper related neurodegenerative diseases [26].

An intriguing link between copper and 5-HT is suggested by symptoms observed in a number of diseases, for instance, in patients with Wilson's disease depression, anxiety, personality changes, and cognitive impairment are among the common symptoms, and a number of studies indicate that disruption of the serotonergic system may partly explain these changes [27,28]. It is well known that serotonin has been implicated in the neurobiology OCD. It has been suggested that OCD might be related to the functioning of brain serotonin systems. This hypothesis is based largely on the notion that SSRIs possess antiobsessional efficacy [29-31]. Neuroimaging studies have been very influential in shaping neurobiological models of OCD. Converging data have implicated a network of brain regions, including the orbitofrontal cortex, striatum, and thalamus, in the pathophysiology of OCD. Most regions of the putatively involved network in OCD are densely innervated by serotonergic or dopaminergic neurons [32]. Also, in animal models and preclinical studies significant interactions between serotonergic and dopaminergic systems in OCD have been reported [33,34].

On the other hand, some in vitro studies have demonstrated that ceruloplasmin is a potent antioxidant, even more potent than albumin and superoxide dismutase [35]. Previous studies suggested an oxidative imbalance in OCD. Selek et al. (2008) reported that total antioxidant status might be increased reactively in OCD [36].

Interestingly, in our study pure OCD patients had a higher mean level of ceruloplasmin than comorbid OCD patients, thus pointing out a more relevant situation between OCD and other disorders. In some schizophrenia studies, plasma ceruloplasmin was associated with subtypes, duration, severity, and antipsychotic treatment of schizophrenia. [3-5,8]. We did not find any association between ceruloplasmin and duration of illness or Y-BOCS-scores of OCD.

Another finding is with a cut-off point of 342.85 U/L ceruloplasmin levels OCD can be predicted in our group. The positive predictive value was 100% and negative predictive value was 91%.

Some limitations of this study include: Sample size is relatively small, not all of the patients have pure OCD, some of them have co-morbid psychiatric disorders, and all patients were receiving drug therapy. The ongoing drug treatments were not interrupted due to ethical considerations.

## Conclusion

To the best of our knowledge, this is the first study about plasma ceruloplasmin in OCD. Although we found that plasma ceruloplasmin was higher in OCD than healthy controls, the role of ceruloplasmin in OCD remains unclear. Our results suggest that alterations in ceruloplasmin levels may reflect a response to illness that serves as some kind of non specific protective mechanism or duration effect of the illness or drug therapy. Further research is warranted to replicate our findings and also to study further the relationship between ceruloplasmin and OCD.

## Abbreviations

OCD: Obsessive Compulsive Disorder; Y-BOCS: Yale-Brown Obsession Compulsion Scale.

## Competing interests

The authors declare that they have no competing interests.

## Authors' contributions

OV drafted the manuscript and performed the analysis and interpretation of the statistical data together with other authors. SS performed to design the study, longitudinal study with respect to OCD in which the subjects of this study are included and the statistical analyses, and has been involved in drafting the manuscript. MB performed longitudinal study with respect to OCD in which the subjects of this study are included together with SS. HC and OE carried out the biochemical examination, performed the analysis and interpretation of the statistical data together with SS. HH and HS has been involved to design the study and the analysis and interpretation of the statistical data. All authors read, provided comments, and approved the final manuscript.
